# Modulation of Phenotype and Function of Human CD4^+^CD25^+^ T Regulatory Lymphocytes Mediated by cAMP-Elevating Agents

**DOI:** 10.3389/fimmu.2016.00358

**Published:** 2016-09-20

**Authors:** Antonella Riccomi, Valentina Gesa, Alessandra Sacchi, Maria Teresa De Magistris, Silvia Vendetti

**Affiliations:** ^1^Department of Infectious, Parasitic and Immune-Mediated Diseases, Istituto Superiore di Sanità, Rome, Italy; ^2^Department of Epidemiology, Preclinical Research, and Advanced Diagnostics, National Institute for Infectious Diseases IRCCS “Lazzaro Spallanzani”, Rome, Italy

**Keywords:** cyclic AMP, cAMP-elevating agents, CTLA-4, costimulation, Treg lymphocytes

## Abstract

We have shown that cholera toxin (CT) and other cyclic AMP (cAMP)-elevating agents induce upregulation of the inhibitory molecule CTLA-4 in human resting CD4^+^ T lymphocytes, which following the treatment acquired suppressive functions. In this study, we evaluated the effect of cAMP-elevating agents on human CD4^+^CD25^+^ T cells, which include the T regulatory cells (Tregs) that play a pivotal role in the maintenance of immunological tolerance. We found that cAMP-elevating agents induce upregulation of CTLA-4 in CD4^+^CD25^−^ and further enhance its expression in CD4^+^CD25^+^ T cells. We observed an increase of two isoforms of mRNA coding for the membrane and the soluble CTLA-4 molecules, suggesting that the regulation of CTLA-4 expression by cAMP is at the transcriptional level. In addition, we found that the increase of cAMP in CD4^+^CD25^+^ T cells converts the CD4^+^CD25^+^Foxp3^−^ T cells in CD4^+^CD25^+^Foxp3^+^ T cells, whereas the increase of cAMP in CD4^+^CD25^−^ T cells did not upregulate Foxp3 in the absence of activation stimuli. To investigate the function of these cells, we performed an *in vitro* suppression assay by culturing CD4^+^CD25^+^ T cells untreated or pre-treated with CT with anti-CD3 mAbs-stimulated autologous peripheral blood mononuclear cell. We found that CT enhances the inhibitory function of CD4^+^CD25^+^ T cells, CD4^+^, and CD8^+^ T cell proliferation and IFNγ production are strongly inhibited by CD4^+^CD25^+^ T cells pre-treated with cAMP-elevating agents. Furthermore, we found that CD4^+^CD25^+^ T lymphocytes pre-treated with cAMP-elevating agents induce the upregulation of CD80 and CD86 co-stimulatory molecules on immature dendritic cells (DCs) in the absence of antigenic stimulation, however without leading to full DC maturation. These data show that the increase of intracellular cAMP modulates the phenotype and function of human CD4^+^CD25^+^ T cells.

## Introduction

Cytotoxic T lymphocyte antigen 4 (CTLA-4) is expressed on T lymphocytes and plays an important role in the down-modulation of immune responses (1, 2). There is accumulating evidence that CTLA-4 plays a dual role, as a mediator of cell-intrinsic negative signals to activated effector T cells and as essential molecule for the function of naturally occurring Foxp3^+^ regulatory T cells (Treg) (3, 4). T regulatory cells constitutively express CTLA-4 (5) and deficiency of the molecule in Foxp3^+^ Tregs impairs their *in vivo* and *in vitro* suppressive function (6–9). Recently, it became evident that CTLA-4 is a negative regulator also on Tregs by limiting their peripheral expansion (10, 11). CTLA-4 is a structural homolog of CD28 and shares with it the ligands CD80 and CD86. However, engagement of CD28 or CTLA-4 delivers opposing signals to T cells, while CD28 promotes IL-2 production and T cell proliferation, CTLA-4 engagement results in impaired IL-2 production and T cell unresponsiveness (12). Furthermore, while CD28 is constitutively expressed on the surface of resting T cells, CTLA-4 is mainly expressed in intracellular recycling vesicles and it is transported to the cell-surface upon T cell activation (13). We have previously shown that cholera toxin (CT) and other cyclic AMP (cAMP) elevating agents induce upregulation of the inhibitory molecule CTLA-4 in human resting CD4^+^ T lymphocytes (14) and that human CD4^+^ T lymphocytes pre-treated with CT inhibit the proliferation of autologous peripheral blood mononuclear cells (PBMC) (15). In this study, we evaluated whether the increase of intracellular cAMP in the absence of stimulation modulate CTLA-4 expression on human CD4^+^CD25^+^ T cells.

Cyclic AMP as a second messenger play a role pivotal in cells of the immune system and this has been widely described (16). The elevation of intracellular cAMP in T lymphocytes has an inhibitory effect on proliferation and on the production of IL-2 by inducing cAMP-dependent ICER expression, which is associated with decreased IL-2 production (17, 18) and has been shown to affect T cell activation events at early stages (19). It has been described that Treg harbor elevated levels of intracellular cAMP that play a role in Treg-mediated suppression by transferring of cAMP to target cells via cell contact-dependent gap junctions (20). Here, we evaluated the phenotype and function of CD4^+^CD25^+^ T cells after treatment with cAMP-elevating agents. We found that increase of intracellular cAMP in CD4^+^CD25^+^ T cells upregulates the inhibitory molecule CTLA-4 and converts CD25^+^ T cells into Foxp3^+^ cells enhancing their suppressive capacity. Furthermore, we observed that in the absence of antigenic stimulation CD4^+^CD25^+^ T cells with increased cAMP levels induce the upregulation of CTLA-4 ligands CD80 and CD86 co-stimulatory molecules on target APC.

## Materials and Methods

### Media and Reagents

RPMI 1640 supplemented with 2 mM l-glutamine, 1% non-essential amino acids, 1% pyruvate, 100 U/ml penicillin, 100 μg/ml streptomycin (Gibco, NY, USA), and 10% FCS (Euroclone, Pero, MI, USA) was used as complete medium in all cultures. Anti-CD3 (clone UCHT1) mAbs were purchased from Immunotech (Westbrook, ME, USA). CT was purchased from List Biological Laboratories (Campbell, CA, USA); forskolin (FSK) was purchased from Sigma Chemicals (Co., St Louis, MO, USA).

### Lymphocytes Purification and Cell Cultures

Human studies were performed in accordance with the ethical guidelines of the 1975 Declaration of Helsinki. PBMC were isolated from freshly collected buffy coats obtained from healthy voluntary blood donors (Bloodbank of University “la Sapienza”, Rome, Italy). The study was approved by the ethical board of our Institute. Cells were isolated by Ficoll-Hypaque (Pharmacia, Sweden) density centrifugation. CD4^+^ T lymphocytes were purified by negative selection using an immunomagnetic cell sorting (Miltenyi Biotec, Bergish Gladback, Germany). CD4^+^ T cells were further purified into CD4^+^CD25^−^ and CD4^+^CD25^+^ T cells by positive selection using phycoerythrin (PE)-anti-CD25 and anti-PE microbeads. Briefly, PBMC were labeled using a cocktail of hapten-conjugated mAbs anti-CD8, CD11b, CD16, CD19, CD36, and CD56 molecules and MACS MicroBeads coupled to an anti-hapten monoclonal antibody. The magnetically labeled cells were depleted by retaining them on a column using MidiMACS cell separator, purity of both CD4^+^CD25^−^ and CD4^+^CD25^+^ T cells was >98%. Alternatively, for mRNA studies CD4^+^CD25^−^ and CD4^+^CD25^+^ T cells were stained with anti-CD4 and anti-CD25 mAbs and isolated by flow cytometry using a BD FACSVantage cell sorter. Purified CD4^+^CD25^+^ T cells (5 × 10^6^/ml) were incubated in the absence or in the presence of CT (1 μg/ml) or FSK (10 μM). After 24 h, cells were harvested, washed extensively (three times), and stained for flow cytometry analysis.

### Flow Cytometry Analysis

Fluorescein (FITC)-, or PE-conjugated anti-CD4, CD8, CTLA-4, CD25, CD127 mAbs and their isotype-matched controls purchased from BD Biosciences, San Diego, CA, USA, and Foxp3 from eBioscience, San Diego, CA, USA, were used for direct immunofluorescence staining. Detection of CTLA-4 and Foxp3 was performed by intracellular staining. Briefly, cells were washed twice in PBS, 1% BSA and 0.1% sodium azide and stained with anti-CD4 or CD25 mAbs on the membrane for 15 min at 4°C. Samples were then fixed in 4% paraformaldehyde for 5 min at 4°C, incubated with anti-CTLA-4 or anti-Foxp3 mAbs, diluted in PBS, 1% BSA and 0.5% saponin. Cells were finally washed twice in PBS, 1% BSA, 0.1% saponin, and acquired on a FACSCalibur™ instrument running CellQuest software.

### CTLA-4 mRNA Analysis by RT-PCR

Total RNA was extracted using RNAFast reagent (Life Technology, Carlsbad, CA, USA) according to the manufacturer’s recommendations. The single-stranded cDNA was synthesized using 1 μg of RNA by reverse transcription using random examers (Invitrogen, Carlsbad, CA, USA). PCRs were performed with cDNA corresponding to 10 ng of RNA, Taq polymerase (Invitrogen, Carlsbad, CA, USA) and primers designed to amplify the entire coding sequence of CD152: 5′-ATGGCTTGCCTTGGATTTCAGCGGCACAAGG-3′ and 5′-TCAATTGATGGGAATAAAATAAGGCTGAAATTGC-3′. PCR was as follows: 94°C for 5 min, 30 cycles 94°C for 30 s, 58°C for 30 s, and 72°C for 30 s followed by a final extension at 72°C for 7 min. The amplified fragments were separated on 1% agarose gel and visualized by ethidium bromide. RNA integrity and cDNA synthesis were verified by amplifying β2-microglobulin cDNA. The intensity of the bands was directly quantified by Image QuaNT software (Amersham Pharmacia Biotech, Piscataway, NJ, USA), which measures a volume report (expressed as arbitrary units, a.u.) by integrating the area of the bands and their OD. CTLA-4 mRNA values were normalized to those of β2-microglobulin and expressed as a.u.

### Proliferation Assay and Cytokines Evaluation

Purified CD4^+^CD25^+^ T cells (5 × 10^6^/ml) were incubated in presence or absence of CT (1 μg/ml). After 24 h, cells were harvested, irradiated (3000 rad), extensively washed, and incubated for 1 h in round-bottom, 96-well plates with autologous PBMC (6 × 10^4^) at different ratios (1:1, 0.5:1, 0.25:1) in triplicates. Then, anti-CD3 mAb (0.5 μg/ml) was added to the cultures, and the proliferation was evaluated by ^3^H-thymidine incorporation. Plates were incubated for 48 h at 37°C with 5% CO2, culture supernatants were collected and frozen at −80°C for cytokine determination by Elisa assay (Pierce Endogen Rockford, IL, USA) and ^3^H-thymidine (Amersham, Aylesbury, UK) was added (1 μCi/well). After 18 h, cells were harvested, and incorporated radioactivity was measured by microβ-counting. Alternatively, PBMC (10^7^/ml) were washed in PBS 1% FCS and incubated with 2.5 μM of carboxyfluorescein succinimidyl ester (CFSE) (Molecular probes, Eugene, OR, USA) for 10 min at 37°C. The reaction was quenched by the addition of 10 times volume of cold RPMI 10% FCS incubated for 5 min at 4°C, then washed three times with RPMI 10% FCS. Cells (8 × 10^4^/well) and co-cultured with CT pre-treated and untreated CD4^+^CD25^+^ T cells (8 × 10^4^/well) and stimulated with anti-CD3 mAbs (0.5 μg/ml). After 4 days, cells were double stained with anti-CD4-PE and anti-CD8-Cy5 mAbs or with anti-CD25-Cy5 and anti-Foxp3-PE and acquired on a FACSCaliburTM instrument running CellQuest software.

### Transwell Experiments

In transwell assays, cells were separated by a membrane (6.5 mm diameter, 0.4 mm pore size) in 24-well plates (Costar, Corning, NY, USA). The lower compartments of the wells contained PBMC (1 × 10^6^). The upper compartments contained medium alone, untreated or CT-pre-treated CD4^+^CD25^+^ T cells (1 × 10^6^). In parallel, untreated or CT-pre-treated CD4^+^CD25^+^ T lymphocytes were placed in the lower chambers in contact with PBMC. Cultures were stimulated with anti-CD3 mAb (0.5 μg/ml) for 48 h, and proliferation was evaluated by harvesting the cells from the lower compartments and by incubating them in the presence of ^3^H-thymidine for further 18 h.

### Monocytes Purification and Cell Cultures

Human monocytes were purified from PBMC of healthy donors by positive selection using anti-CD14-conjugated magnetic microbeads (Miltenyi Biotec, Bergisch Gladbach, Germany). Cells recovered were 95–99% CD14^+^, as determined by flow cytometry using the FITC conjugate anti-human CD14 mAb (BD Biosciences, San Diego, CA, USA). Monocyte-derived DCs were generated by culturing isolated monocytes for 5 days in complete RPMI medium with 35 ng/ml rhIL-4 and 50 ng/ml rhGM-CSF (Immunological Sciences, Rome, Italy). Immature DCs were co-cultured for 48 h with CD4^+^CD25^+^ T cells pre-treated or untreated with CT or in presence of lipopolysaccharide (LPS) (200 ng/ml) from Sigma Chemical Co, CT, USA (1 μg/ml) or FSK 10 μM). Cells were stained with FITC-, PE-, or PECy7-conjugated anti-HLA-DR, anti-HLA-A,B,C, anti-CD80, anti-CD83 and anti-CD86 mAbs. All samples were acquired on a FACSCaliburTM instrument running CellQuest software.

### Statistical Analysis

Microsoft Excel (Microsoft Corp., Redmond, WA, USA) was used for statistical analysis. Data were expressed as mean ± SEM, and statistical significance was determined by Student’s *t*-test, *p* ≤ 0.05 was considered statistically significant.

## Results

### Cyclic AMP Upregulates CTLA-4 Molecules in CD4^+^CD25^−^ and in CD4^+^CD25^+^ T Lymphocytes

**Figure 1:**
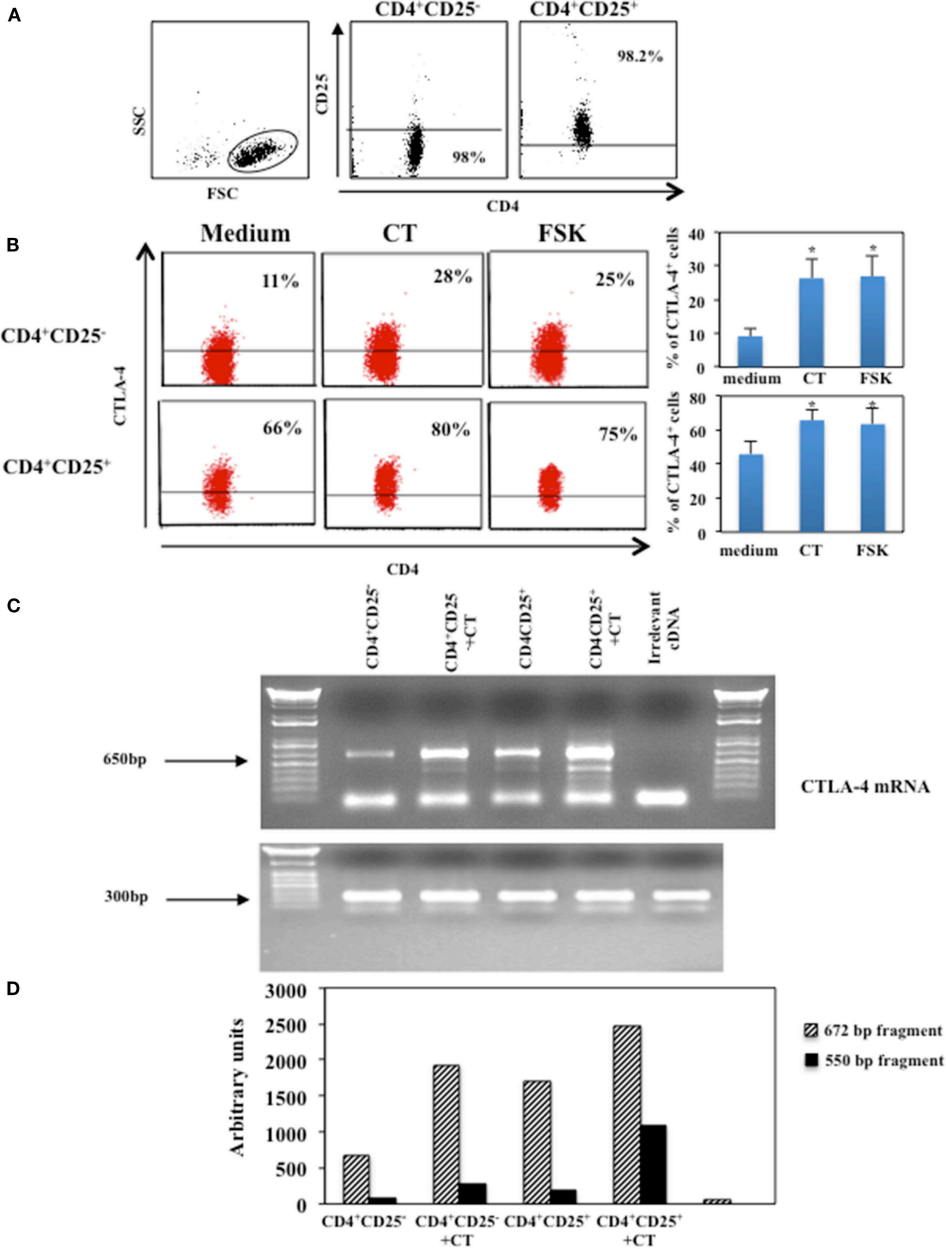
**Upregulation of CTLA-4 induced by cAMP in the absence of T cell activation**. CD4^+^CD25^−^ and CD4^+^CD25^+^ T cells were purified by immunomagnetic cell sorting, **(A)** shows the gate strategy and purity of cells after magnetic separation. Purified cells were cultured in the presence of medium alone (medium), CT (1 μg/ml) or FSK (10 μM) for 24 h and then stained with anti-CD4 mAb on the membrane, fixed, permeabilized and stained with anti-CTLA-4. Cytofluorimetric analysis was performed on CD4^+^-gated population. Graphs show the mean of the upregulation of CTLA-4 of five independent donors and the *indicates that the differences between CT or FSK treated as compared with untreated cells are significant (*p* < 0.05), the variations bars correspond to the SEM **(B)**. CTLA-4 mRNA was evaluated in CD4^+^CD25^−^ and CD4^+^CD25^+^ T lymphocytes purified by FACSvantage cell sorting after staining with anti-CD25 mAb. Cells were cultured in the presence or absence of CT and the mRNA was analyzed after 4 h. RNA integrity and cDNA synthesis were verified by amplifying β2-microglobulin cDNA. An irrelevant cDNA from human tumor cell line (THP-1) has been included in the amplification (lane 6) of **(C)**. The graph **(D)** represents CTLA-4 mRNA quantification of the two mRNA transcripts (672 and 550 bp fragments), both normalized to those of β2-microglobulin. One of two experiments performed is shown.

We have shown that CT and other cAMP elevating agents induce upregulation of the inhibitory molecule CTLA-4 in human resting CD4^+^ T lymphocytes (14). In this study, to investigate whether intracellular increase of cAMP affects on the expression of CTLA-4 on CD4^+^CD25^+^ T cells, we evaluated the effects of cAMP-elevating agents on CD4^+^CD25^−^ and CD4^+^CD25^+^ T lymphocytes. Cells were purified by immunomagnetic cell sorter, the purity of the CD4^+^CD25^−^ and CD4^+^CD25^+^ T cells and the gate strategy is shown in Figure 1A and cultured in the presence and in the absence of CT (1 μg/ml) or FSK (10 μM), a drug that directly activates adenylyl cyclases, for 24 h and analyzed for the CTLA-4 expression by intracellular staining. We found that the increase of intracellular cAMP enhances the expression of CTLA-4 on both CD4^+^CD25^−^ and CD4^+^CD25^+^ T cells (Figure 1B). Although it is known that CD4^+^CD25^+^ Tregs constitutively express CTLA-4 and that its expression is upregulated following activation (5, 10, 21), the treatment with cAMP-elevating agents further enhances its expression in CD4^+^CD25^+^ T cells in the absence of activation stimuli (Figure 1B).

Next, we evaluated whether cAMP-dependent upregulation of CTLA-4 correlated with an increase of mRNA, we analyzed the CTLA-4 mRNA in CD4^+^CD25^−^ and CD4^+^CD25^+^ T cells purified by FACSvantage cell sorting after staining with anti-CD25 mAb. Cells were cultured in the presence of CT and the mRNA was analyzed after 4 h. We observed an increase of two mRNA transcripts corresponding to the membrane (672 bp) and the soluble (550 bp) molecule of CTLA-4 (22) (Figures 1C,D). These results show that the increase of intracellular cAMP results in the upregulation of soluble and membrane CTLA-4 at mRNA and protein levels in T lymphocytes and that this occurs in the absence of full T cell activation.

### Cyclic AMP Elevating Agents Induce the Expression of Foxp3 in CD4^+^CD25^+^ T Lymphocytes

**Figure 2:**
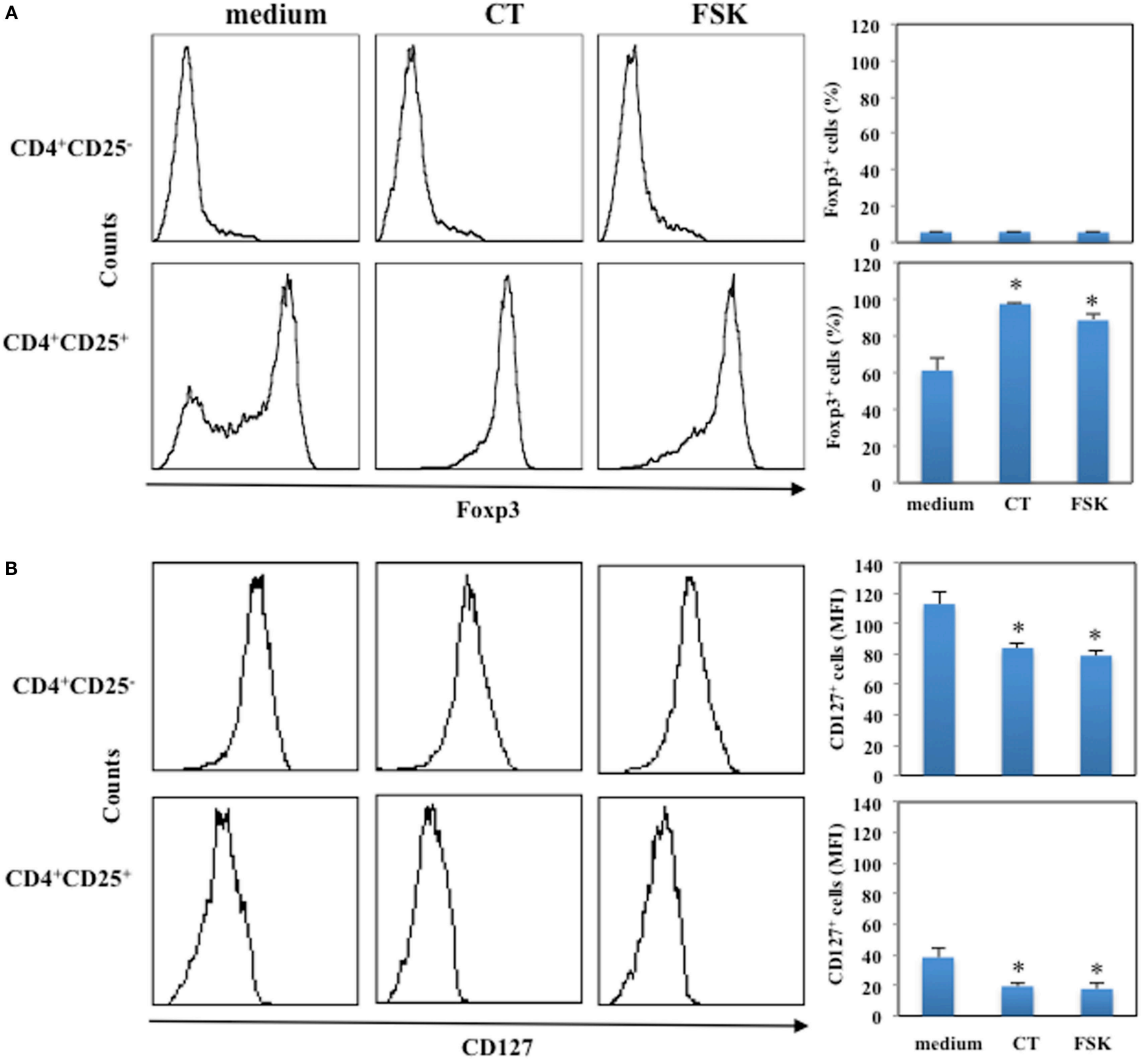
**Upregulation of Foxp3 induced by cAMP in the absence of T cell activation**. CD4^+^CD25^−^ and CD4^+^CD25^+^ T cells were purified by immunomagnetic cell sorter, cultured in the presence of medium alone (medium), CT (1 μg/ml) or FSK (10 μM) for 24 h and stained with anti-CD4 and anti-CD127 mAb **(B)** or with anti-CD4 and on membrane and the fixed, permeabilized and stained with anti-Foxp3. Cytofluorimetric analysis was performed on CD4^+^-gated population. Graphs show the mean of the upregulation of Foxp3 **(A)** and down-modulation of CD127 **(B)** of five independent donors and the * indicates that the differences between CT or FSK treated as compared with untreated cells are significant (*p* < 0.05).

Next, we asked whether the increase of intracellular cAMP could affect the expression of Foxp3 in CD4^+^CD25^−^ and in CD4^+^CD25^+^ T lymphocytes. Cells were purified by immunomagnetic cell sorter, cultured in the presence and in the absence of CT (1 μg/ml) or FSK (10 μM) for 24 h and analyzed for Foxp3 expression by intracellular staining. The percentage of Foxp3^+^ cells among CD4^+^CD25^+^ T lymphocytes was evaluated and was 61 ± 7%. We found that the increase of intracellular cAMP by either CT or FSK enhances the expression of Foxp3 on CD4^+^CD25^+^ T cells, whereas it did not change Foxp3 expression on CD4^+^CD25^−^ T cells (Figure 2A). Furthermore, we observed a significant down-modulation of CD127 following CT or FSK treatment on both CD4^+^CD25^−^ and on CD4^+^CD25^+^ T cells (Figure 2B). These data indicate that the increase of cAMP in CD4^+^CD25^+^ T cells converts the CD4^+^CD25^+^Foxp3^−^ T cells in CD4^+^CD25^+^Foxp3^+^ Tregs, whereas the increase of cAMP in CD4^+^CD25^−^ T cells did not upregulate Foxp3 in the absence of activation stimuli.

### Enhanced Suppression of T Cell Proliferation by CD4^+^CD25^+^ T Lymphocytes Pre-Treated with CT

**Figure 3:**
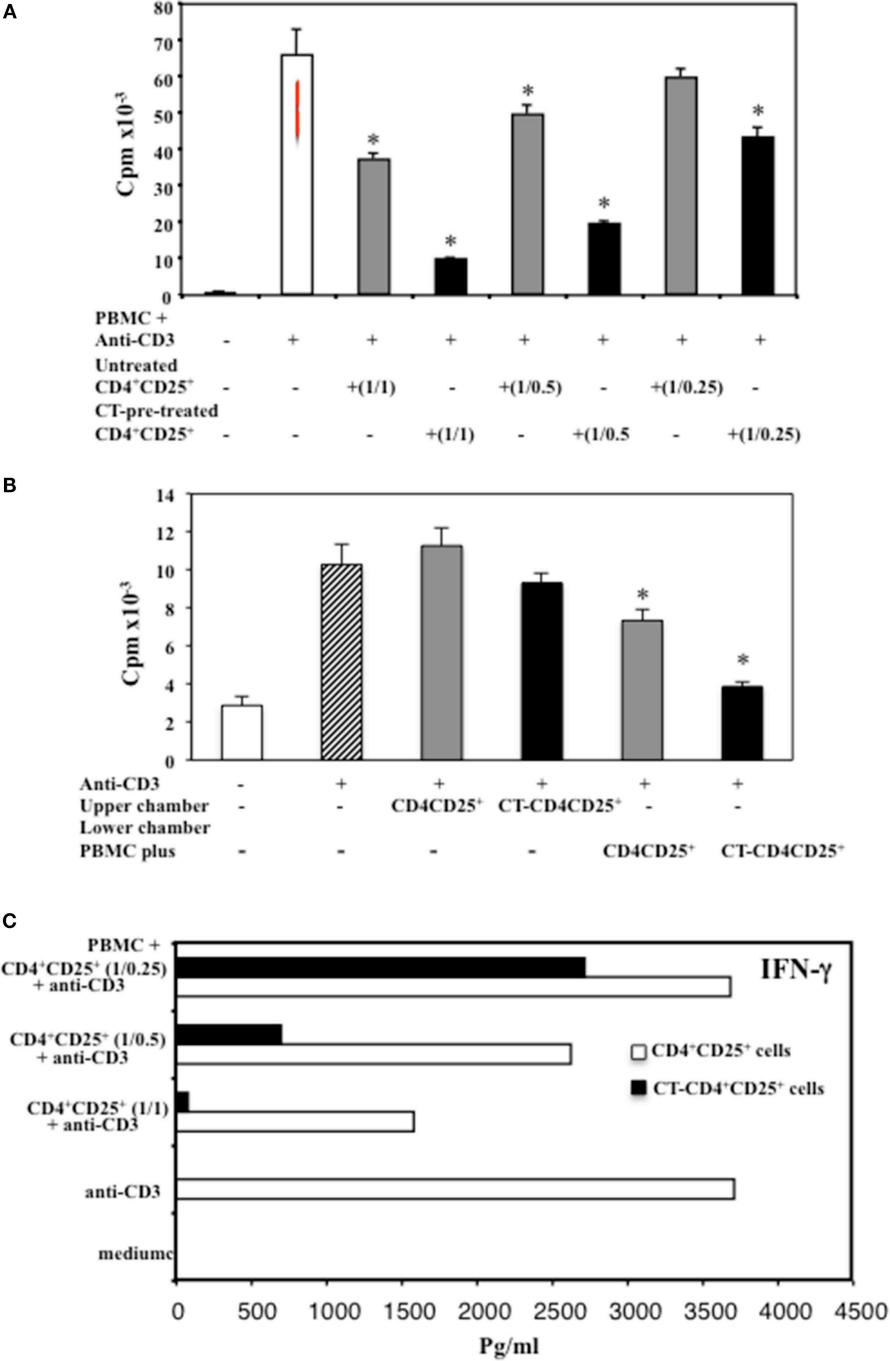
**Enhanced suppression of T cell proliferation and IFN**γ **inhibition by CD4^+^CD25^+^ T lymphocytes pre-treated with CT**. **(A)** Immunomagnetically purified CD4^+^CD25^+^ T lymphocytes isolated from healthy donors were cultured in the presence or absence of CT (1 μg/ml). After overnight incubation, cells were washed three times and cultured with autologous PBMC at different ratios (1/1, 1/0.5, 1/0.25) in triplicate in 96-well plates and stimulated with anti-CD3 mAbs. **(B)** Alternatively, PBMC (1 × 10^6^) were placed in the lower wells of transwell and untreated or CT-pre-treated CD4^+^CD25^+^ T lymphocytes (1 × 10^6^) were cultured either in the upper wells or in the lower chambers in contact with PBMC. Cells were stimulated with anti-CD3 mAb (0.5 μg/ml), and the PBMC proliferation was evaluated after 66 h by ^3^H-thymidine incorporation. The bars indicate the mean of triplicate wells. **(C)** After 48 h, the amount of IFNγ was valuated in the supernatants from the different cultures by ELISA assay in duplicate. The * indicate that the differences between CT-treated as compared with untreated cells are significant (*p* < 0.05). One representative for each of three experiments performed is shown.

In order to analyze the function of CD4^+^CD25^+^ T lymphocytes pre-treated with cAMP-elevating agents, we compared the suppressive capacity of untreated and CT pre-treated CD4^+^CD25^+^ T cells. PBMC isolated from healthy donors were cultured with autologous untreated or CT-pre-treated CD4^+^CD25^+^ T cells at different ratios (1/1, 1/0.5, 1/0.25) and stimulated with anti-CD3 mAbs (0.5 μg/ml). The proliferation was evaluated by ^3^H-thymidine incorporation after 66 h of culture. According to the higher proportion of Foxp3^+^ T cells in the CT-pre-treated CD4^+^CD25^+^ T lymphocytes, we found that CD4^+^CD25^+^ T lymphocytes pre-treated with CT showed a higher capacity of inhibiting the proliferation of autologous PBMC as compared to untreated CD4^+^CD25^+^ T cells. The inhibition was dose dependent (Figure 3A). To investigate whether the suppression mediated by CT-pre-treated CD4^+^CD25^+^ T lymphocytes was cell-to-cell contact dependent, we performed experiments using transwell. PBMC were placed in the lower chambers of the transwell and untreated or CT-pre-treated CD4^+^CD25^+^ T lymphocytes in the upper chambers. In parallel, untreated or CT-pre-treated CD4^+^CD25^+^ T lymphocytes were placed in the lower chambers in contact with PBMC. Cultures were stimulated with anti-CD3 mAb. We found that CT-pre-treated CD4^+^CD25^+^ T cells inhibited the proliferation of autologous, anti-CD3-stimulated PBMC in cell-to-cell contact (*n* = 3, *p* < 0.05), although the inhibition was slightly higher when PBMC and CT-pre-treated CD4^+^CD25^+^ T lymphocytes were placed in the upper chambers, the differences were not significant (Figure 3B). These results suggest that the suppression induced by CT-pre-treated CD4^+^CD25^+^ T cells is mediated by cell-to-cell contact mechanism.

**Figure 4:**
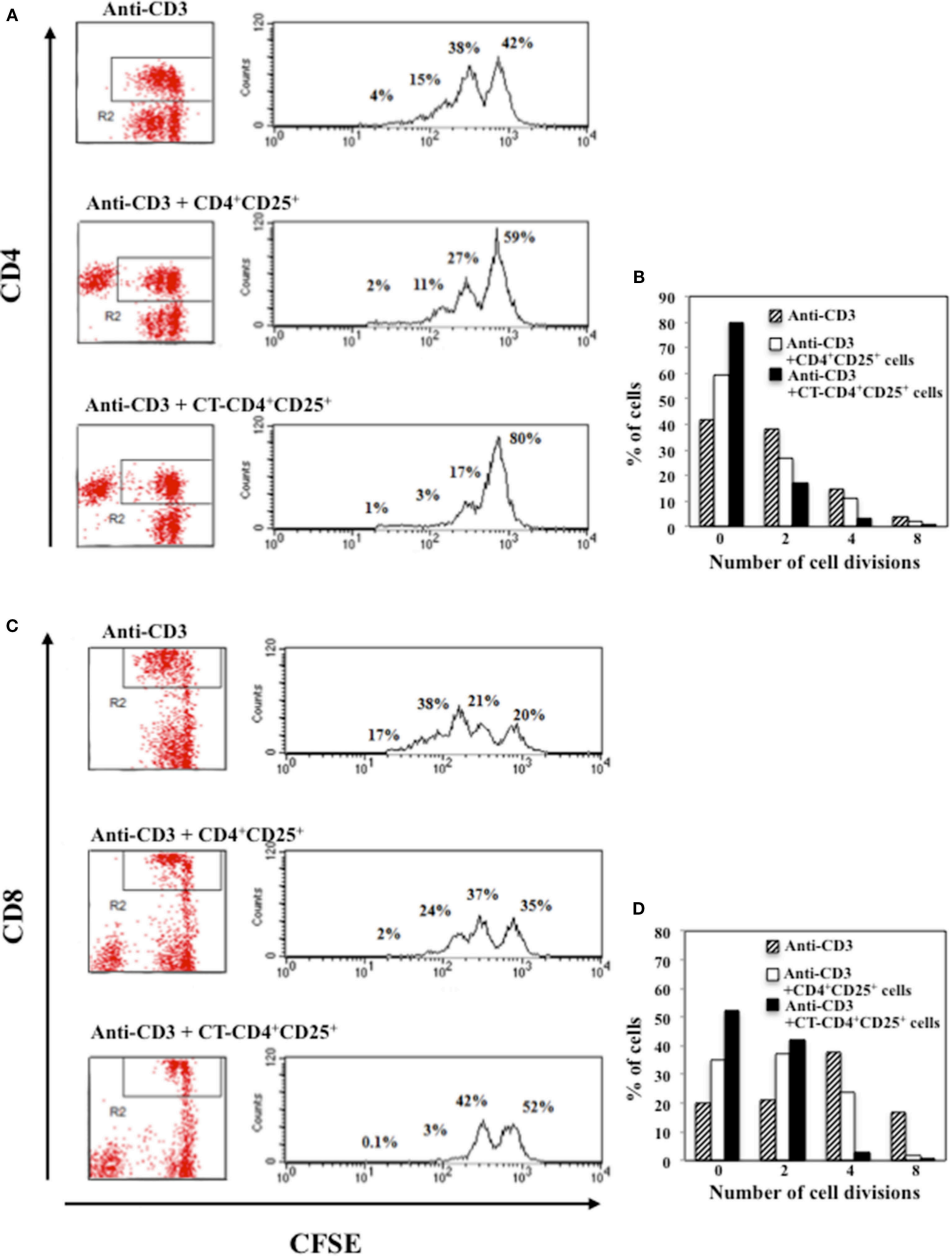
**Inhibitory effects of CD4^+^CD25^+^ T lymphocytes pre-treated and untreated with CT on CD4 or CD8 T cell proliferation**. Immunomagnetically purified CD4^+^CD25^+^ T lymphocytes isolated from healthy donors were cultured in the presence or absence of CT (1 μg/ml). After overnight incubation, cells were washed three times and cultured with autologous CFSE-labeled PBMC at 1/1 ratio and stimulated with anti-CD3 mAbs. The percentage of proliferating CD4^+^ **(A,B)** and CD8^+^ **(C,D)** T cells was evaluated by FACS analysis after 5 days of culture by CFSE dilution on CD4 and on CD8-gated populations. The numbers within the plots and the graphs indicate the percentage of cells that went through 0, 2, 4, and 8 cell divisions. One representative of three performed experiments is shown.

The production of IFNγ was evaluated in the supernatants after 48 h of culture and we found that CD4^+^CD25^+^ T lymphocytes pre-treated with CT and stimulated with anti-CD3 inhibited the production of IFNγ in the co-cultures with autologous PBMC in a dose-dependent manner, and at larger extent as compared to untreated CD4^+^CD25^+^ T cells (Figure 3C). To further evaluate the inhibitory effects of CD4^+^CD25^+^ T lymphocytes treated and untreated with CT on either CD4 or CD8 T cell proliferation, PBMC were labeled with CFSE and cultured with autologous untreated or CT-pre-treated CD4^+^CD25^+^ T cells at 1/1 ratio and stimulated with anti-CD3 mAbs (0.5 μg/ml). The percentage of proliferating CD4^+^ T cells was evaluated by FACS analysis after 5 days of culture. Figure 4 shows that both CD4 and CD8 T lymphocytes go through a less number of divisions when they are co-cultured with CT-pre-treated CD4^+^CD25^+^ T lymphocytes than with untreated cells as shown by the progressive halving of CFSE in dividing cells. These data indicate that CD4^+^CD25^+^ T lymphocytes with increased cAMP levels have an increased suppression capacity as compared to untreated cells.

### Enhanced Suppression of CD4^+^CD25^+^ T Lymphocytes Pre-Treated with CT Correlates with Higher Foxp3 Expression in the Co-Cultures

**Figure 5:**
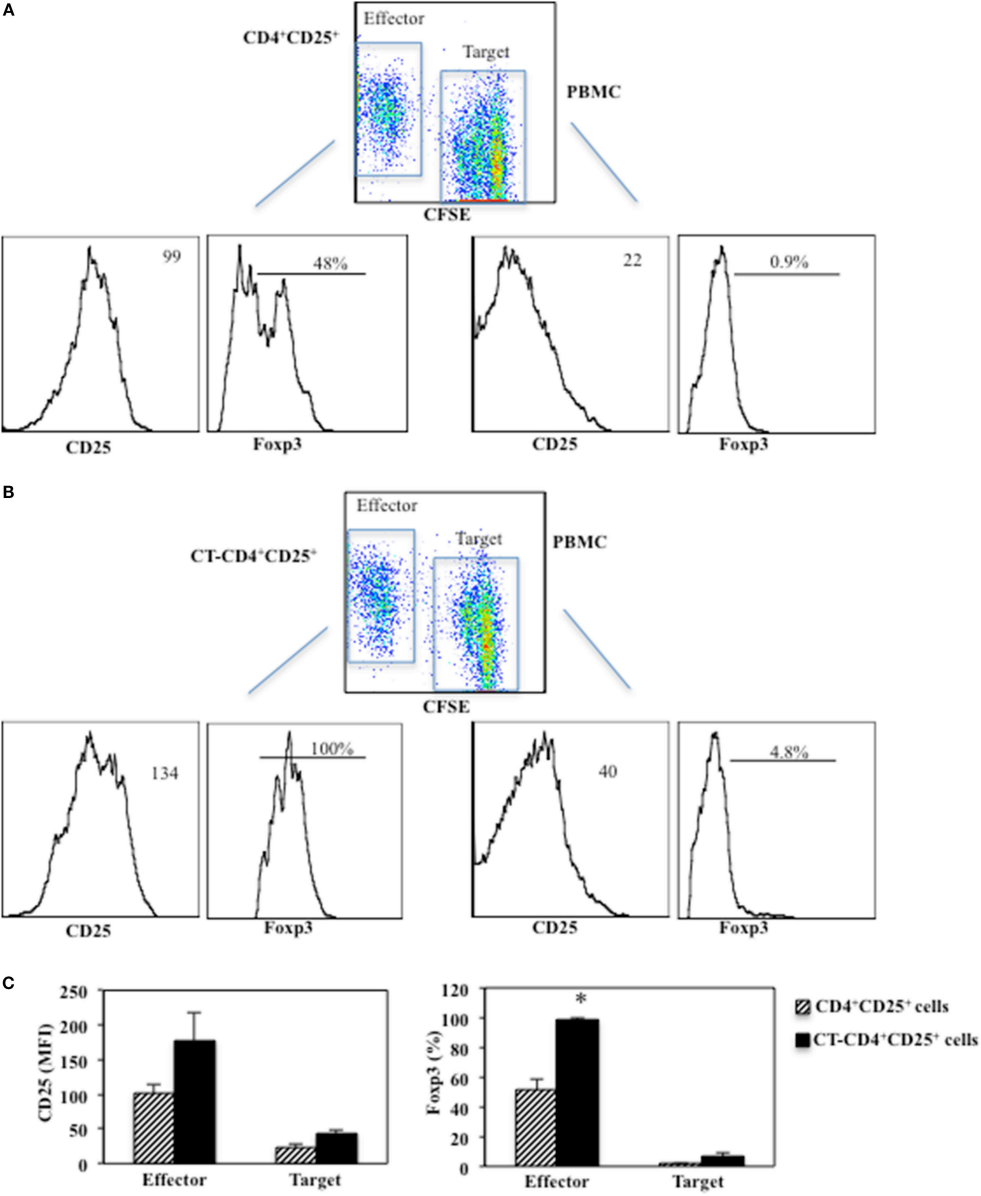
**Enhanced suppression of CD4^+^CD25^+^ T lymphocytes pre-treated with CT correlates with higher Foxp3 expression**. Immunomagnetically purified CD4^+^CD25^+^ T lymphocytes isolated from healthy donors were cultured in the presence or absence of CT (1 μg/ml). After overnight incubation, CD4^+^CD25^+^ T lymphocytes treated **(B)** or untreated with CT **(A)** (called Effector cells) and CFSE-labeled PBMC (called Target cells) cells were cultured at 1/1 ratio and stimulated with anti-CD3 mAbs. The proliferation was measured as CFSE dilution in Target cells and the expression of CD25 and Foxp3 was evaluated by FACS analysis after 5 days of culture on both effector and target cells. Graphs **(C)** show the mean fluorescence intensity (MFI) values of CD25 and the percentage of Foxp3^+^ cells in Effector and Target cells of three independent experiments and the * indicate that the differences between untreated and CT-pre-treated CD4^+^CD25^+^ T cells are significant (*p* < 0.05).

Next, we evaluated the expression of CD25 and Foxp3 after 5 days of co-cultures between CD4^+^CD25^+^ T lymphocytes treated or untreated with CT (called Effector cells) and CFSE-labeled PBMC (called Target cells). Target cells were labeled with CFSE and cultured with autologous untreated or CT-pre-treated CD4^+^CD25^+^ T cells at 1/1 ratio and stimulated with anti-CD3 mAbs (0.5 μg/ml). The proliferation was measured as CFSE dilution and the expression of CD25 and Foxp3 was evaluated by FACS analysis after 5 days of culture. We found that CD25 was upregulated on both Effector (178 ± 39 vs. 101 ± 12.5 mfi, *p* = 0.04) and Target cells (43.2 ± 4.1 vs. 23.3 ± 6.1 mfi, *p* = 0.009) when the Effector cells were CT-pre-treated CD4^+^CD25^+^ T lymphocytes as compared to when untreated CD4^+^CD25^+^ T cells were present in the cultures (Figure 5). On the other hand, Foxp3 was upregulated on CT-pre-treated CD4^+^CD25^+^ T lymphocytes as compared to untreated CD4^+^CD25^+^ T lymphocytes (98.5 ± 1% vs. 51.7 ± 7.7%, *p* = 0.017) and in a small percentage of the target cells co-cultured with CT-pre-treated T lymphocytes as compared to target cells co-cultured with untreated cells (6.4 ± 3.1% vs. 1.6 ± 0.9%, *p* = 0.09). These data once again show that the higher suppression exerted by CT-pre-treated CD4^+^CD25^+^ T lymphocytes correlates with the higher expression of Foxp3.

### CD4^+^CD25^+^ T Lymphocytes Pre-Treated with cAMP-Elevating Agents Induce the Upregulation of CD80 and CD86 on Immature DCs in the Absence of Antigenic Stimulation

**Figure 6:**
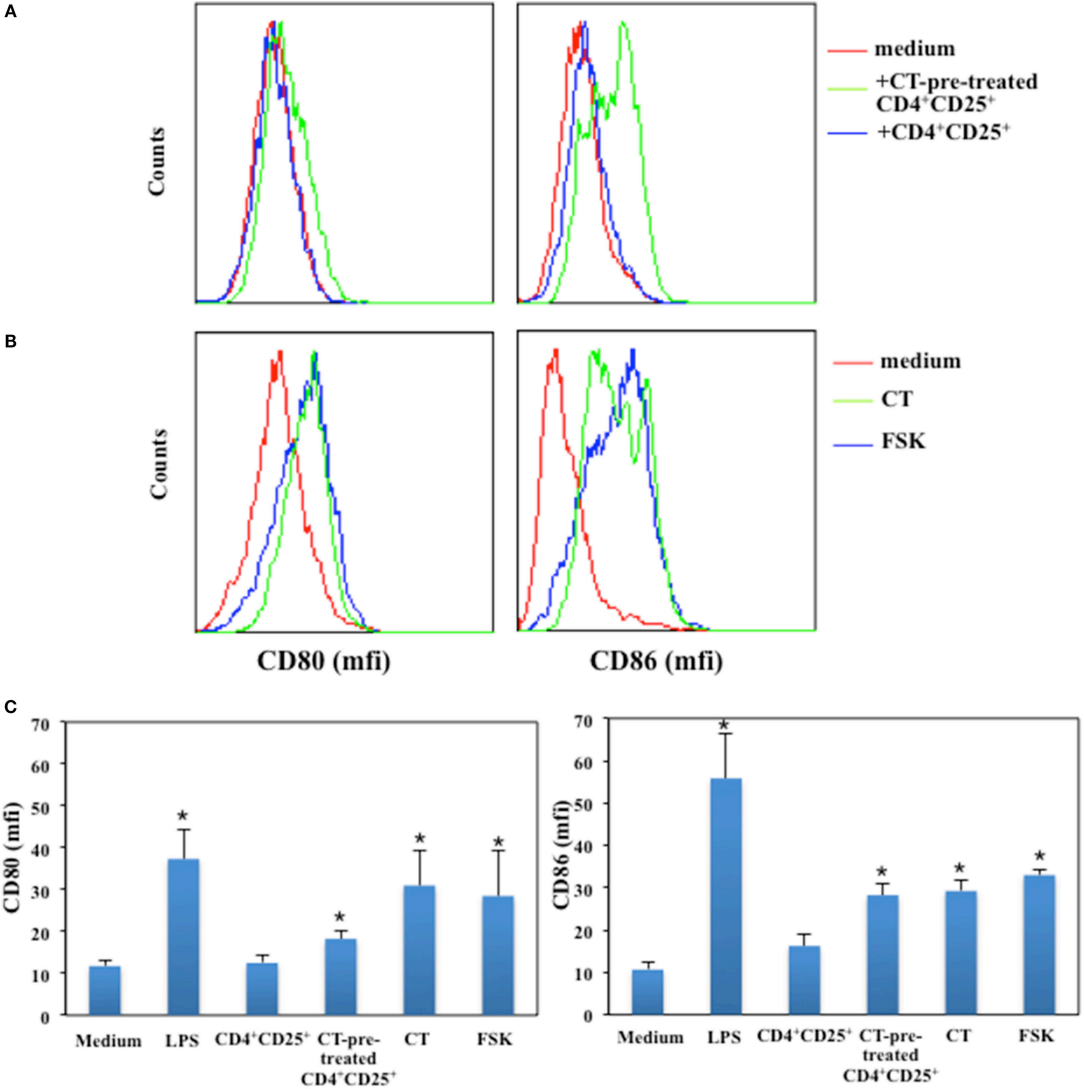
**Upregulation of CD80 and CD86 on immature DCs by CD4^+^CD25^+^ T lymphocytes pre-treated with cAMP-elevating agents**. Immature DCs were generated by culturing peripheral human monocytes isolated from healthy donors with GM-CSF and IL-4 for 5 days. In parallel, autologous CD4^+^CD25^+^ T lymphocytes were purified and cultured in presence and absence of CT for 24 h. After have been extensively washed CD4^+^CD25^+^ T lymphocytes were co-cultured with immature DCs for further 48 h. Dendritic cells cultured with LPS (200 ng/ml) **(A,C)**, CT or FSK **(B)** were included as control. The expression of CD80 and CD86 co-stimulatory molecules on DCs was evaluated by FACS analysis. Graphs in panel **(C)** show the mean of the CD80 and CD86 expression of five independent donors and the * indicates that the differences as compared to untreated cells are significant (*p* < 0.05).

It has been reported that CD4^+^CD25^+^ T cells affect DCs functions and maturation (23) and that this is due together with other molecular mechanisms also to the constitutive expression of CTLA-4 on their membrane, which by interacting with its ligands leads to DCs suppression (6, 24). Therefore, we asked whether CD4^+^CD25^+^ T cells pre-treated or untreated with a cAMP-elevating agents were able to modulate the expression of co-stimulatory molecules on immature DCs in the absence of antigenic stimulation. Immature DCs were generated by culturing peripheral human monocytes isolated from healthy donors with GM-CSF and IL-4 for 5 days. In parallel, autologous CD4^+^CD25^+^ T lymphocytes were purified and cultured in presence and absence of CT for 24 h. After have been extensively washed, CD4^+^CD25^+^ T lymphocytes were co-cultured with immature DCs for further 48 h. Dendritic cells (DCs) cultured with LPS were included as control to evaluate DC maturation. The expression of CD80 and CD86 co-stimulatory molecules on DCs was evaluated by FACS analysis. We found that CD4^+^CD25^+^ T lymphocytes pre-treated with CT induced the upregulation of both CD80 and CD86 co-stimulatory molecules on DCs as compared to untreated CD4^+^CD25^+^ T cells, which did not alter the expression of the co-stimulatory molecules in the absence of maturation stimuli (Figures 6A,C). However, when we analyzed the expression of MHC class I and II and that of CD83, a specific maturation marker of DCs, we found that CT-pre-treated CD4^+^CD25^+^ T cells did not change their expression (Table 1). On the other hand, LPS induced the upregulation of these markers as expected. These results show that CD4^+^CD25^+^ T lymphocytes with increased cAMP levels induce the upregulation of co-stimulatory molecules on DCs without leading to full maturation of DCs. In parallel, the effects of cAMP-elevating agents, such as CT and FSK, on DC maturation were evaluated as control. We found that both CT and FSK led to a significant upregulation of CD80 and CD86 molecules (Figures 6B,C). In the same experiments, although we observed a significant upregulation of MHC class I and II and that of CD83 by CT, with FSK we observed a significant upregulation only of MHC class II and not of MHC class I and CD83 (Table 1). These data suggest that the increase of cAMP, unless is not sustained like when is induced by CT, lead to partial DC maturation. Altogether, these findings indicate that the molecular effects of the cAMP on the immune responses are the result from the integration of inhibitory as well as co-stimulatory stimuli on different cell types.

**Table 1:** **Expression of different maturation markers on DCs co-cultured with untreated or CT-pre-treated CD4^+^CD25^+^ T cells**.

	Medium	CD4^+^CD25^+^	CT-pre-treated CD4^+^CD25^+^	CT	FSK	LPS
HLA-I	16 ± 7	18.5 ± 4.9	18.5 ± 3.5	21.5 ± 0.7	19.1 ± 0.2	32 ± 7.5*
HLA-DR	68 ± 7	68 ± 1	69.5 ± 7.7	131 ± 4.2*	81 ± 4.9*	135.2 ± 14.3*
CD80	11.7 ± 1.2	12.5 ± 2.6	18.7 ± 2.9*	31 ± 8.4*	28.8 ± 10.6*	37.3 ± 6.86*
CD86	10.6 ± 1.8	16.3 ± 3.9	28.2 ± 3.8*	29.2 ± 2.5*	33 ± 1.4*	55.8 ± 10.7*
CD83	4.5 ± 1.3	5.5 ± 0.7	6.5 ± 0.7	7.1 ± 0.8*	5.73 ± 1.06	17 ± 4.4*

Human CD14 monocytes, isolated from healthy donors’ PBMC, cultured for 5 days with GM-CSF (50 ng/ml) and IL-4 (35 ng/ml) were cultured with medium alone, untreated or CT-pre-treated CD4^+^CD25^+^ T cells or with LPS (200 ng/ml), CT (1 μg/ml) or FSK (10 μM) for 48 h. Cells were stained using directly conjugated mAbs and analyzed by flow cytometry. The data are the mean of the expression of these indicated markers of four independent donors and bars are SEM, * indicates that the differences between the indicated treatment and the ones cultured in medium alone are significant (*p* < 0.05).

## Discussion

The integration of co-stimulatory and inhibitory signals is a critical aspect of the immune responses. CTLA-4 is an inhibitory receptor expressed on T lymphocytes, which is normally upregulated upon T cell activation and is involved in the termination of immune responses. On the other hand, the natural ligands of CTLA-4, CD80, and CD86 co-stimulatory molecules are upregulated following APC activation and maturation (25). We have shown that CT and other cAMP-elevating agents induce upregulation of the inhibitory molecule CTLA-4 in human resting CD4^+^ T lymphocytes (14) and that these cells with increased intracellular cAMP levels exert inhibitory function by releasing cAMP (15). Since CTLA-4 plays a critical role in cell contact-dependent Treg-mediated *in vitro* and *in vivo* suppression (6, 21), we asked whether signals that lead to an increase of intracellular cAMP would modulate CTLA-4 expression in CD4^+^CD25^+^ T cells. It is known that Tregs constitutively express CTLA-4 and that its expression is upregulated in the presence of activation stimuli (5, 10, 21). Here, we show that cAMP-elevating agents enhance the expression of this inhibitory molecule in CD4^+^CD25^−^ and further upregulate its expression in CD4^+^CD25^+^ T cells. These data are in accordance with others showing that different cAMP-elevating agents, such as adenosine or β-adrenergic agonists, induce CTLA-4 upregulation in Treg (26–28). We observed increased CTLA-4 mRNA in cells treated with CT. It is known that two mRNA isoforms coding for soluble and membrane CTLA-4 are expressed in resting T lymphocytes (22). Interestingly, the upregulation of human CTLA-4 by cAMP-elevating agents in CD4^+^CD25^+^ T lymphocytes involves both variants of mRNA transcripts. The upregulation of CTLA-4 by cAMP could be regulated at the transcriptional level or due to changes in the stability of CTLA-4 mRNA. Although the contribution of sCTLA-4 to immune regulation has been less studied, it has been reported to play a role as regulator of T cell responses and to potentiate Treg suppression functions (29–31). These findings are consistent with the enhanced suppression of cytokine production and T cell proliferation on both CD4^+^ and CD8^+^ T cell populations exerted by CD4^+^CD25^+^ T lymphocytes pre-treated with CT. Furthermore, consistent with the higher suppression capacity mediated by CD4^+^CD25^+^ T cells pre-treated with CT as compared with untreated cells is the increased percentage of CD4^+^CD25^+^Foxp3^+^ found after treatment with CT or FSK. This is in agreement with other findings showing that signaling through A2A adenosine or through β-adrenergic receptors, which leads to an increase of intracellular cAMP, induces the expression of Foxp3 on CD4^+^Foxp3^−^ cells (26–28). Indeed, the Foxp3 gene was described to bear an enhancer in its first intron that is dependent on a cAMP response element binding protein (CREB) site (32). In addition, our results indicate that the increase of cAMP in CD4^+^CD25^+^ T cells converts the CD4^+^CD25^+^Foxp3^−^ T cells into CD4^+^CD25^+^Foxp3^+^ Tregs, whereas the increase of cAMP in CD4^+^CD25^−^ T cells did not upregulate Foxp3 in the absence of activation stimuli following 24-h treatment. These data suggest that the presence of CD4^+^CD25^+^Foxp3^+^ cells in the cultures is needed for the upregulation of Foxp3 mediated by cAMP-elevating agents. Although the molecular mechanisms underlying the upregulation of CTLA-4 and Foxp3 in CD4^+^CD25^+^ T cells it is not completely understood, at this stage, we can assess by using the CT, which constitutively increases the intracellular cAMP and FSK, which only transiently increases cAMP, that these effects are mediated by cAMP and that a single shot of cAMP, which *in vivo* could be caused by different factors, such as neurotransmitters or catecholamine, can change the phenotype and functions of CD4^+^CD25^+^ T cells. Increase of cAMP activates downstream pathways that are mainly the canonical PKA and the non-canonical EPAC pathways. The cAMP/PKA signaling leads to the phosphorylation of the CREB, which has been described to promote the TGFβ-mediated generation of Tregs from naive T cells and contribute to the expression of Foxp3 in these induced Tregs. In light of this, we can hypothesize that the upregulation of CTLA-4 and Foxp3 on CD4^+^CD25^+^ T cells are mediated by PKA/CREB pathway, however, further studies are needed to fully clarify the molecular mechanism.

Cyclic AMP has been regarded to mediate Treg suppression (20). It was demonstrated that cAMP is transferred from Tregs into activated target responder T cells via gap junctions (20). It was also shown that direct contact between Tregs and conventional T cells can induce ICER expression in responder T cells by cAMP-dependent mechanism, which is associated with down-modulation of IL-2 production (20, 33–36). Furthermore, targeting ICER with RNAi in responder CD4^+^ T cells led to the inhibition of Treg-mediated suppression (34–36). In addition, we have previously shown that CD4^+^ T lymphocytes treated with cAMP-elevating agents release cAMP in the extracellular compartment in the absence of cell death (15) and that extracellular cAMP could be sensed by cells of the immune system through CD73 ecto-5′-nucleotidase and A2A and A2B adenosine receptors (37). Furthermore, it is known that Tregs express high levels of ecto-enzymes CD73 and CD39, which generate extracellular adenosine (38–40) and adenosine through the A2A adenosine receptor pathway, not only potentiate the suppression function of Treg but also promote their expansion (26, 27, 40–42). Therefore, we can hypothesize that extracellular cAMP could be converted into adenosine through the extracellular cAMP-adenosine pathway, which has been described in different cell types included cells of the immune system (37) and acts on the effector cells through the adenosine receptors. This could in part explain the need of CD4^+^CD25^+^Foxp3^+^ Tregs, which express high levels of CD73 to speed up the conversion of CD4^+^CD25^+^Foxp3^−^ T cells into CD4^+^CD25^+^Foxp3^+^ Tregs in the presence of cAMP-elevating agents. On the other hand, intracellular cAMP could be transferred to the target cells through the gap junctions and inhibit T cell proliferation or modulate APC function. These two mechanisms are not necessary mutually exclusive and both lead to an increase of intracellular cAMP in target cells. Consistent with this are the effects mediated by CD4^+^CD25^+^ T lymphocytes pre-treated with CT on DCs. In line with our previous observations (43, 44), the increase of cAMP in immature DC in the absence of maturation stimuli, unless is constitutive such as that induced directly by CT, leads to moderate upregulation of CD80 and CD86 co-stimulatory molecules on DCs and did not change the expression of MHC class I and II and that of CD83, a specific DC maturation marker as compared to treatment with LPS, which induced full maturation of DCs. On the other hand, it has been reported that CD4^+^CD25^+^ T cells affect DCs functions and maturation (23, 24) by down-modulating CD80 and CD86 through CTLA-4, which by interacting with its ligands leads to DCs alteration and sequestration (6, 23, 24). Our data, which could apparently be in contrast with these findings could be explained by taking into account that the effect of CT pre-treated CD4^+^CD25^+^ T cells on DCs was observed in the absence of maturation stimuli and antigenic stimulation, reminding the effects of cAMP on immature DCs (43, 44). The upregulation of co-stimulatory molecules on APC could concur to a stronger interaction between Treg and APC and further prevent them to interact with and stimulate responder T cells. On the contrary, it is known that the capacity to increase cAMP is an important feature of several adjuvants, which strongly stimulate the immune responses (45). We have reported that even if cAMP impairs the differentiation of monocytes into DCs, it induces professional myeloid APC (46). Therefore, the overall role of cAMP on the immune responses according with its inhibitory and co-stimulatory stimuli on different cell types remains to be further investigated. This concept was recently reviewed by Raker et al. (47).

In conclusion, we show that cAMP plays different roles according to cell types upregulates CTLA-4 and Foxp3 on CD4^+^CD25^+^ T cells in the absence of activation stimuli and Tregs pre-treated with cAMP-elevating agents induce the upregulation of CD80 and CD86 on APC. The manipulation of intracellular and extracellular cAMP could be a tool for therapeutic purposes in autoimmune and inflammatory diseases.

## Author Contributions

All authors listed have made substantial, direct, and intellectual contribution to the work and approved it for publication.

## Conflict of Interest Statement

The authors declare that the research was conducted in the absence of any commercial or financial relationships that could be construed as a potential conflict of interest.

## Abbreviations

cAMP, cyclic AMP; CT, cholera toxin; FSK, forskolin.
